# Comparative Genomics of Two ST 195 Carbapenem-Resistant *Acinetobacter baumannii* with Different Susceptibility to Polymyxin Revealed Underlying Resistance Mechanism

**DOI:** 10.3389/fmicb.2015.01445

**Published:** 2016-01-05

**Authors:** Soo-Sum Lean, Chew Chieng Yeo, Zarizal Suhaili, Kwai-Lin Thong

**Affiliations:** ^1^Faulty of Science, Institute of Biological Sciences, Universiti MalayaKuala Lumpur, Malaysia; ^2^Faculty of Medicine, Biomedical Research Centre, Universiti Sultan Zainal AbidinKuala Terengganu, Malaysia; ^3^Faculty of Bioresources and Food Industry, Universiti Sultan Zainal AbidinKuala Terengganu, Malaysia

**Keywords:** *Acinetobacter baumannii*, carbapenem-resistant, polymyxin, whole genome sequencing, resistance island, resistance mechanisms

## Abstract

*Acinetobacter baumannii* is a Gram-negative nosocomial pathogen of importance due to its uncanny ability to acquire resistance to most antimicrobials. These include carbapenems, which are the drugs of choice for treating *A. baumannii* infections, and polymyxins, the drugs of last resort. Whole genome sequencing was performed on two clinical carbapenem-resistant *A. baumannii* AC29 and AC30 strains which had an indistinguishable *Apa*I pulsotype but different susceptibilities to polymyxin. Both genomes consisted of an approximately 3.8 Mbp circular chromosome each and several plasmids. AC29 (susceptible to polymyxin) and AC30 (resistant to polymyxin) belonged to the ST195 lineage and are phylogenetically clustered under the International Clone II (IC-II) group. An AbaR4-type resistance island (RI) interrupted the *comM* gene in the chromosomes of both strains and contained the *bla*_OXA−23_ carbapenemase gene and determinants for tetracycline and streptomycin resistance. AC29 harbored another copy of *bla*_OXA−23_ in a large (~74 kb) conjugative plasmid, pAC29b, but this gene was absent in a similar plasmid (pAC30c) found in AC30. A 7 kb Tn*1548*::*armA* RI which encodes determinants for aminoglycoside and macrolide resistance, is chromosomally-located in AC29 but found in a 16 kb plasmid in AC30, pAC30b. Analysis of known determinants for polymyxin resistance in AC30 showed mutations in the *pmrA* gene encoding the response regulator of the two-component *pmrAB* signal transduction system as well as in the *lpxD, lpxC*, and *lpsB* genes that encode enzymes involved in the biosynthesis of lipopolysaccharide (LPS). Experimental evidence indicated that impairment of LPS along with overexpression of *pmrAB* may have contributed to the development of polymyxin resistance in AC30. Cloning of a novel variant of the *bla*_AmpC_ gene from AC29 and AC30, and its subsequent expression in *E. coli* also indicated its likely function as an extended-spectrum cephalosporinase.

## Introduction

*Acinetobacter baumannii* is a significant nosocomial pathogen that infects immunocompromised patients, including patients with underlying diseases, especially those who are warded in intensive care units (ICU; Bergogne-Bérézin and Towner, 1996; Nordmann and Poirel, 2008; Camp and Tatum, 2010). These nosocomial infections include ventilator-associated pneumonia, secondary meningitis, endocarditis, urinary tract infections, surgical site infections, and blood stream infections (Camp and Tatum, 2010; Huang et al., 2012). *A. baumannii* is intrinsically resistant to commonly used antibiotics such as aminopenicillins, first- and second-generation cephalosporins and chloramphenicol (Dijkshoorn et al., 2007). The notoriety of this pathogen stems from its ability to develop and acquire resistance to almost all antimicrobial drugs as well as its tolerance to desiccation and ability to survive on inanimate surfaces for prolonged periods of time (Camp and Tatum, 2010; Roca et al., 2012; Doi et al., 2015). In depth analyses of the resistance mechanisms in *A. baumannii* revealed that its multidrug resistance phenotype is mediated by all the major resistance mechanisms that are known to occur in bacteria, including modification of target sites, enzymatic inactivation, active efflux, and decreased influx of drugs (Dijkshoorn et al., 2007).

Carbapenem resistance in *A. baumannii* is usually acquired with the most significant mechanism being the production of carbapenemases (Poirel and Norman, 2006). *A. baumannii* naturally produces the OXA-51-group carbapenemase at a low level. The transposition of an insertion sequence (usually IS*Aba1* or IS*Aba9*) upstream of the *bla*_OXA−51_ gene provides a strong promoter for expression of the carbapenemase leading to elevation of carbapenem MICs and thus, resistance (Higgins et al., 2010). *A. baumannii* also readily acquires several OXA-group β-lactamases usually through transposons and plasmids with OXA-23 being the most prevalent (Poirel and Norman, 2006; Roca et al., 2012).

*A. baumannii* naturally produces the AmpC-type β-lactamase and overexpression of the *bla*_AmpC_ gene has been associated with the presence of IS*Aba1* providing a strong promoter leading to cephalosporin resistance (Segal et al., 2005; Tian et al., 2011). These *Acinetobacter*-derived cephalosporinases (ADCs) typically hydrolyze penicillins, narrow- and extended-spectrum cephalosporins but not cefepime or carbapenems (Rodríguez-Martínez et al., 2010). Recently, several ADC variants have been identified that are capable of hydrolyzing cefepime and these are termed as extended-spectrum AmpCs (ESACs) (Rodríguez-Martínez et al., 2010; Tian et al., 2011).

It is of great concern when carbapenems, which are the drugs of choice for treatment, are increasingly compromised. As the efficacy of these drugs decreases, polymyxins were re-introduced as the drug of “last resort” (Landman et al., 2008). The renewed interest in polymyxins (polymyxin B and colistin, or polymyxin E) as therapeutic agents was due to the pathogen's outer membrane, polyanionic lipopolysaccharide (LPS) having high affinity toward the cationic polymyxin molecules (Landman et al., 2008). Binding of the LPS and polymyxin molecules resulted in a detergent-like effect that disrupts the outer membrane thereby causing cytoplasm leakage of the pathogen (Landman et al., 2008; Arroyo et al., 2011). Although polymyxins were once discarded due to their neurotoxicity and nephrotoxicity, polymyxins have a more effective antibacterial activity against most Gram negative pathogens, including *A. baumannii* (Landman et al., 2008). Previous studies from Garnacho-Montero et al. (2003) and Moffatt et al. (2010) showed that intravenous polymyxins were safe to use as an effective treatment to *Acinetobacter* infections. However, uncontrolled use or overuse of polymyxins in the hospital environment may lead to the development of polymyxin-resistance in *A. baumannii* (Arroyo et al., 2011).

Polymyxin resistance in *A. baumannii* appeared to develop intrinsically as a result of exposure to this class of drugs. Two major mechanisms of polymyxin resistance have been described for *A. baumannii*. The first is the modification of the lipid A moiety of LPS with phosphoethanolamine as a result of mutations in the *pmrA/pmrB* two-component signal transduction system which leads to the up-regulated expression of the *pmrCAB* operon. Overexpression of *pmrC* which encodes the enzyme responsible for phosphoethanolamine addition to lipid A, impairs the binding of polymyxin to the outer membrane thereby leading to resistance (Adams et al., 2009; Arroyo et al., 2011; Beceiro et al., 2011; Park et al., 2011). The second mechanism is the complete loss of the LPS caused either by mutations or the insertional inactivation of the lipid A biosynthesis genes, namely *lpxA, lpxC*, and *lpxD* (Moffatt et al., 2010, 2011). Mutations in the *lpsB* gene that encodes a glycosyltransferase involved in the biosynthesis of the LPS core have also been implicated in polymyxin resistance (Hood et al., 2013).

We have previously characterized 54 *A. baumannii* strains obtained from a tertiary hospital in Terengganu, Malaysia (Lean et al., 2014). Out of these, 39 were carbapenem- and multidrug-resistant (MDR). Among the 39 carbapenem resistant strains, 14 were also resistant to polymyxin B and categorized as extensive-drug resistant (XDR). Two strains, *A. baumannii* AC29 and AC30 were isolated from the wounds of different patients, and shared an identical *Apa*I pulsotype. However, AC29 is susceptible to polymyxin B whereas AC30 was resistant with an MIC value of 128 μg/ml. Here, we report the comparative genomic analyses of these two *A. baumannii* strains, AC29 and AC30, to show that despite sharing an identical pulsotype, there are significant changes in the genome structure particularly in the resistance islands and the plasmid content of the two isolates. We also present experimental evidence to elucidate possible mechanisms for the development of polymyxin resistance in *A. baumannii* AC30 and the likely implication of a novel *bla*_AmpC_ cephalosporinase gene in resistance to extended-spectrum cephalosporins as well as imipenem in both AC29 and AC30.

## Materials and methods

### Whole genome sequencing

#### Strains selection, antibiotic resistance profiles, and DNA extraction

Two strains of *A. baumannii* AC29 and AC30 from a tertiary hospital in Terengganu, Malaysia were selected for this study (Lean et al., 2014). Both strains were obtained from the wounds of different patients using standard microbiology procedures. *A. baumannii* AC29 and AC30 shared an identical *Apa*I pulsotype and showed resistance to gentamycin, tobramycin, amikacin, ciprofloxacin, levofloxacin, piperacillin-tazobactam, cefotaxime, ceftazidime, cefepime, ampicillin-sulbactam, tetracycline, and doxycycline. Both isolates were also resistant to carbapenem with MIC values of >32 μg/ml for both imipenem and meropenem (Clinical and Laboratory Standards Institute, 2013). However, AC29 was susceptible to polymyxin B whereas AC30 was resistant with an MIC value of 128 μg/ml (Magiorakos et al., 2011; Lean et al., 2014).

Genomic DNA of AC29 and AC30 were extracted using Wizard Genomic DNA Purification Kit (Promega, USA) according to the manufacturer's instructions. Extracted DNA was quantified using the spectrophotometer at OD_260_ and the purity was determined by OD_260_/OD_280_ ratio (Ausubel et al., 2002).

### Genomic sequencing, assembly, and annotations

Whole genome sequencing of *A. baumannii* AC29 and AC30 was carried out by a commercial vendor using the Illumina Genome Analyzer IIx platform. CLC Bio software package was used to assemble the genome sequence data. Open reading frame (ORF) prediction and gene functional assignments were done using Prodigal 2.60 (Hyatt et al., 2010), RNAmmer 1.2 (Lagesen et al., 2007), and tRNAscan-SE (Lowe and Eddy, 1997). Functional annotation of the genome was performed using Blast2Go and the Rapid Annotation using Subsystem Technology (RAST) server (Aziz et al., 2008).

### MLST and phylogenetic analysis

To determine the sequence types, multilocus sequence typing (MLST) of AC29 and AC30 was performed according to the Bartual or Oxford scheme using the seven housekeeping genes *cpn60, gdhB, gltA, gpi, gyrB, recA*, and *rpoD* (Bartual et al., 2005) and the Pasteur scheme using the genes *cpn60, fusA, gltA, pyrG, recA, rplB*, and *rpoB* (Diancourt et al., 2010). The gene sequences were compared to the PubMLST database for *A. baumannii* (http://pubmlst.org/abaumannii/) and assigned to the appropriate sequence types.

The phylogenetic relationship of AC29 and AC30 to sequenced *A. baumannii* strains was inferred using composition vector tree (CVTree) version 2 (Xu and Hao, 2009) based on the concatenated nucleotide sequences of the seven reference genes used in the Bartual MLST scheme. The CVTree web-based server (http://tlife.fudan.edu.cn/cvtree/) generates phylogenetic trees based on *k*-tuple values (Xu and Hao, 2009). The completed *A. baumannii* genomes used in the analysis were downloaded via the NCBI FTP site and the reference gene sequences extracted for phylogenetic analysis.

### Comparative genomics analyses

Whole genome sequences of these two *A. baumannii* AC29 and AC30 strains were compared to our previously reported *A. baumannii* AC12 genome (Lean et al., 2015) and the completed *A. baumannii* genomes available from NCBI FTP site using Mauve (Darling et al., 2004). Circular chromosome and plasmid map of these strains were constructed using CGView Server developed by the Stothart Research Group (Grant and Stothard, 2008).

#### Accession numbers

Genome sequences of *A. baumannii* strains AC29 and AC30 were deposited under the accession number CP007535 and CP007577, respectively. Plasmid sequences of pAC29a, pAC29b, pAC30a, pAC30b, and pAC30c were deposited under the accession number CP008850, CP008851, CP007578, CP007579, and CP007580, respectively. Accession numbers for our previously reported *A. baumannii* strain AC12 (CP007549) and pAC12 (CP007550) were as in (Lean et al., 2015).

### Quantitative real-time PCR of *pmrAB*

One of the gene loci that have been implicated in polymyxin resistance is the *pmrCAB* operon with *pmrAB* encoding the two-component signal transduction system and *pmrC* encoding the enzyme that catalyzes the addition of phosphoethanolamine to the lipid A moiety of lipopolysaccharide (LPS; Arroyo et al., 2011; Park et al., 2011; Hood et al., 2013). To determine the transcript levels of *pmrAB* from the polymyxin-susceptible AC29, and the polymyxin-resistant isolates AC30 as well as AC12 (Lean et al., 2015), quantitative real-time PCR (qRT-PCR) was employed. *A. baumannii* ATCC19606 was used as the polymyxin-susceptible control. Total RNA was extracted from *A. baumannii* strains grown in LB broth using Qiagen RNeasy Mini Kit (Qiagen).

The extracted total RNA was subjected to reverse transcription using the Quantitect Reverse Transcription Kit (Qiagen) in a T-Gradient PCR machine from Biometra/Applied Biosystems. The resulting cDNA produced was used as template for quantitative real-time PCR using the SYBR Green PCR Kit (Qiagen) performed in a Rotor-Gene 6000 Real-Time PCR Machine (Corbett Life Science/Qiagen). Prior to the actual amplification cycling conditions, the mixture was subjected to initial heat activation at 95°C for 5 min followed by 35 cycles of the two-step cycling condition of denaturation at 95°C for 10 s and annealing at 60°C for 30 s. The melting curve profile for each amplification reaction and the relevant C_T_ value were automatically generated using the software provided (Corbett Life Science/Qiagen). The *rpoB* gene was used as the housekeeping gene for normalization (Park et al., 2011). Relative quantification using the ΔΔC_T_ method (Pfaffl, 2001) was then applied to quantify the expression of *pmrAB*.

### Lipopolysaccharide analysis

Lipopolysaccharide (LPS) analysis was carried out for the polymyxin resistant strains AC30 and AC12 (Lean et al., 2015), the polymyxin susceptible strain AC29 along with *A. baumannii* ATCC19606 as the polymyxin-susceptible control. LPS extraction was carried out according to Hood et al. (2013). Briefly, bacteria were swabbed from LB agar plates into 154 mM NaCl, and the cell density was adjusted to an OD_600_ of 1.5, pelleted and resuspended in lysis buffer (2% SDS, 4% 2-mercaptoethanol, 10% glycerol, 0.1 M Tris-HCl, pH 6.8). Samples were then boiled for 10 min, cooled to 60°C and treated with proteinase K for 1 h. These samples were electrophoresed through a 15% polyacrylamide gel using standard methods and stained with Pro-Q Emerald 300 LPS stain according to the manufacturer's recommendations (Invitrogen). Molecular weight marker used in the experiment was the Precision Plus Protein Dual Xtra Standards (BioRad). The stained minigels were then viewed and photographed under UV light using a UV Gel Imager (Alpha-Innotech).

#### Cloning and expression of the novel variant *bla*_AmpC_ gene

The novel variant *bla*_AmpC_ gene was PCR-amplified from the genomes of *A. baumannii* AC29 and AC30 using specific primers ampC_BamHI: 5′-GGATCCATGGCTGTGGGTGT TATTCAA-3′ and ampC_HindIII: 5′-AAGCTTTTATTTCTTT ATTGCATTCAGCAC-3′ with the following conditions—initial hold at 95°C for 3 min, followed by 40 cycles of 95°C for 50 s, 55°C for 50 s, 72°C for 1 min, and final extension at 72°C for 90 s. Purified PCR products were then cloned into pGEM-T Easy (Promega, USA) according to the manufacturer's instructions and transformed into *E. coli* JM109. Transformants were selected on LB agar supplemented with 100 mM ampicillin with 100 mM IPTG and 50 mg/ml X-gal. Plasmids were prepared from white colonies and screened by digestion with *Bam*HI and *Hin*dIII which cut at the restriction sites incorporated into the primers used for amplification. Plasmids with the expected restriction fragments were validated by conventional Sanger dideoxy sequencing prior to subcloning into the pET30a expression vector. The resulting pET30a-*ampC* recombinant plasmids were then transformed into *E. coli* BL21 (DE3/pLysS) and expression of the cloned *bla*_AmpC_ gene carried out by inducing the cells with 0.1 mM IPTG. After that, the *E. coli* BL21 recombinant clones were tested for their MIC values for extended-spectrum cephalosporins (i.e., ceftazidime and cefepime) as well as aztreonam and imipenem at concentrations of 2, 4, 8, 16, and 32 μg/ml using the agar dilution method.

## Results

### Strain characteristics and genome analyses

#### Basic genome features and sequence types

Analyses of the whole genome sequencing indicated that the *A. baumannii* AC29 and AC30 genomes were 3,935,134 and 3,925,274 bp, with GC content of 38.84 and 38.98%, respectively. Predicted ORFs from the genomes of *A. baumannii* AC29 and AC30 were 3728 and 3646, respectively. Both strains contained only one chromosome with varying number of plasmids, as featured in Table 1. The Bartual or Oxford scheme for MLST utilizes seven housekeeping genes *cpn60, gdhB, gltA, gpi, gyrB, recA*, and *rpoD* to determine the sequence type (ST) of *A. baumannii* (Bartual et al., 2005). Both AC29 and AC30 were designated ST195 (derived from *cpn60*-2, *gdhB*-3, *gltA*-1, *gpi*-96, *gyrB*-3, *recA*-2, and *rpoD*-3) whereas using the Pasteur MLST scheme (Diancourt et al., 2010), both *A. baumannii* strains were designated ST2 (*cpn60*-2, *fusA*-2, *gltA*-2, *pyrG*-2, *recA*-2, *rplB*-2, and *rpoB*-2). Thus, AC29 and AC30 were categorized as belonging to the International Clone-II (or IC-II, also referred to as Global Clonal 2, or GC2) group of strains.

**Table 1:** **General genomic features of the whole genome sequences of *A. baumannii* strains AC29 and AC30**.

**Feature**	**Strains**	
	**AC29**	**AC30**
Accumulated length	3,935,134 bp	3,925,274 bp
Average GC content	38.84%	38.98%
Number of contigs	102	91
Number of ORF	3728	3646
Number of tRNA	66	58
Number of rRNA	4	3
Number of plasmid	2	3
Plasmid size	pAC29a (8737 bp) pAC29b (74,749 bp)	pAC30a (8729 bp) pAC30b (16,236 bp) pAC30c (71,433 bp)

**Figure 1:**
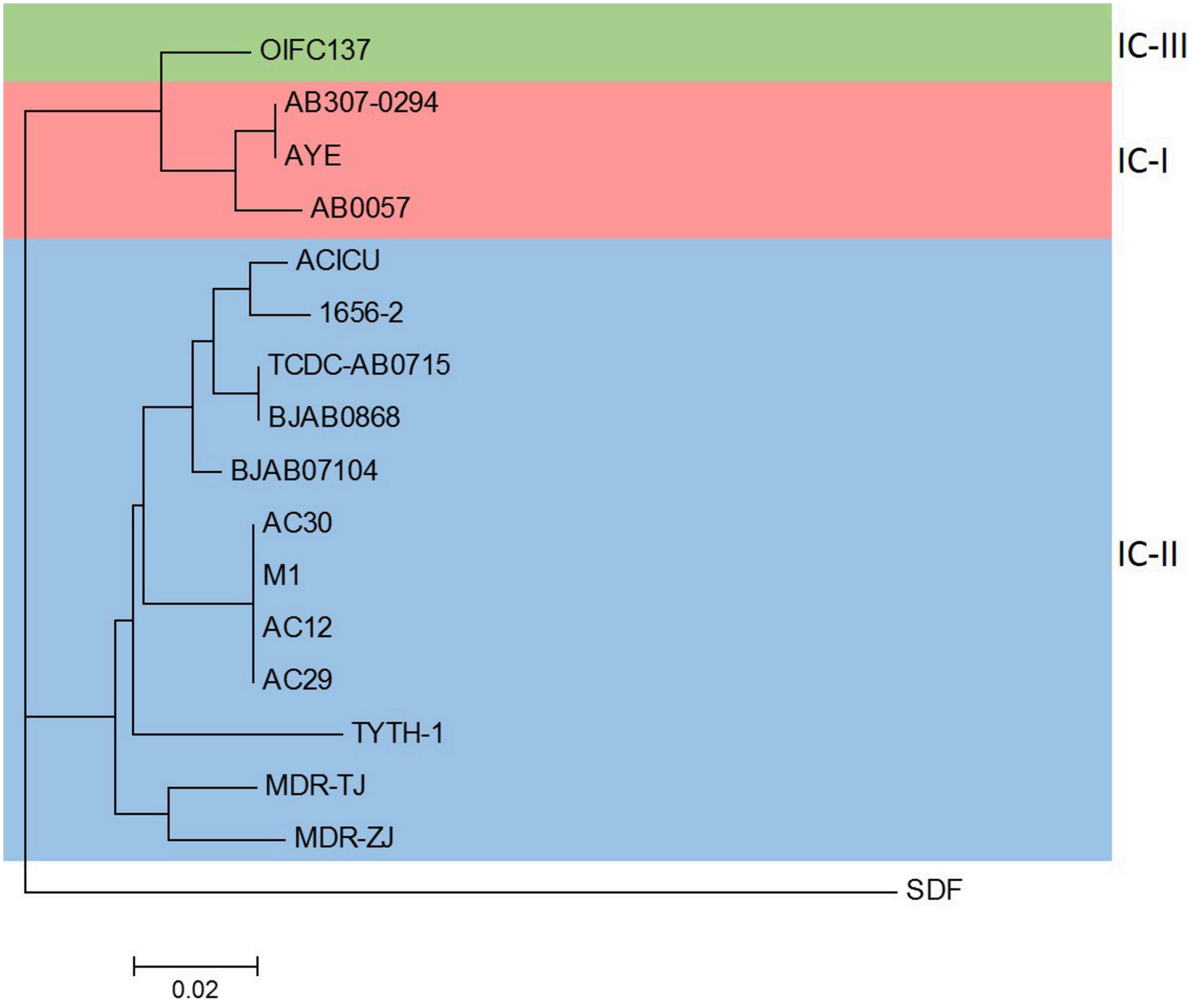
**Phylogenetic analysis of *A. baumannii* AC12, AC29, AC30, and completed *A. baumannii* genomes obtained from NCBI**. The phylogenetic tree was constructed by composition vector tree version 2 (CVTree 2) using concatenated nucleotide sequences of the seven reference genes based on the Bartual/Oxford MLST scheme (*cpn60, gdhB, gltA, gpi, gyrB, recA*, and *rpoD*). Strains belonging to the international clonal groups IC-I, IC-II, and IC-III are indicated. The accession nos. of the strains used in the analysis are as follows: AB307-0294 (CP001172.1), AYE (CU459141.1), AB0057 (CP001182.1), ACICU (CP000863.1), 1656-2 (CP001921.1), TCDC-AB0715 (CP002522.2), BJAB0868 (CP003849.1), BJAB07104 (CP003846.1), M1 (LAIL01000001.1), TYTH-1 (CP003856.1), MDR-TJ (CP003500.1), MDR-ZJ (CP001937.1), OIFC137 (NZ_AFDK01000002.1), SDF (NC_010400.1).

Phylogenetic analysis of the *A. baumannii* AC29 and AC30 genomes were performed with other available *A. baumannii* genomes from the NCBI's FTP site and using *A. baumannii* SDF as an outgroup to root the phylogenetic tree based on the concatenated nucleotide sequences of the seven reference genes utilized in the Bartual MLST scheme (Farrugia et al., 2013). Based on the phylogenetic tree (Figure 1), *A. baumannii* AC29 and AC30 were closely related to *A. baumannii* AC12 which was also isolated from the same hospital in Terengganu and was also typed as ST195 (Lean et al., 2015) along with *A. baumannii* M1 which was also isolated from Malaysia (accession no. LAIL0100000). These strains were clustered together with other *A. baumannii* isolates of the IC-II group and this was also reflected in their genome grouping based on genomic BLAST (as indicated at http://www.ncbi.nlm.nih.gov/genome/genomegroups/403? accessed on 19 August 2015).

### The AbaR4-type resistance island

**Figure 2:**
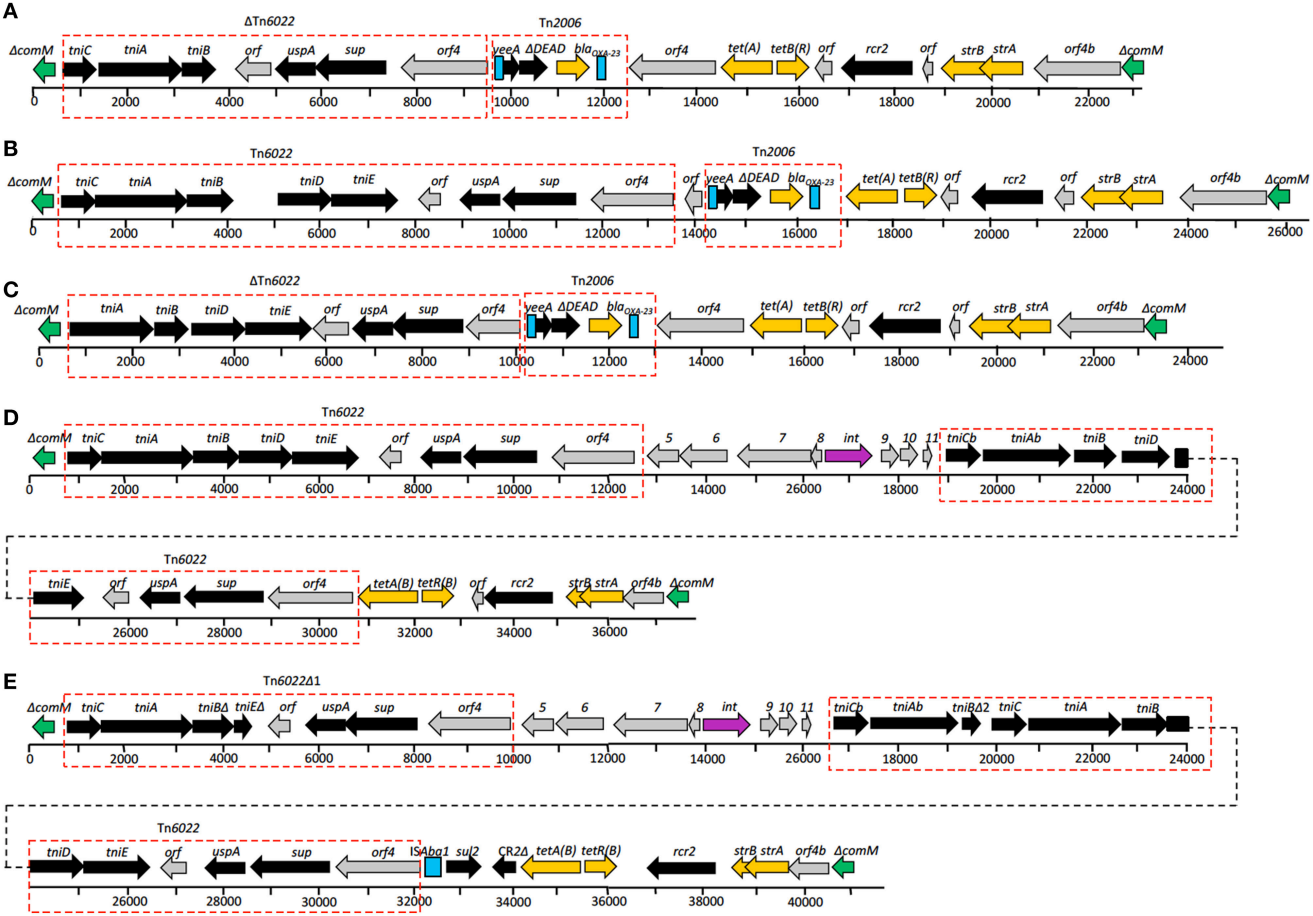
**Structures of resistance islands present in *A. baumannii* AC12, AC30, and AC29 as compared to similar islands in *A. baumannii* MDR-ZJ and MDR-TJ**. **(A)** Structure of resistance island ACRI12-1 present in *A. baumannii* AC12. ΔTn*6022*, Tn*6022*, and Tn*2006* are indicated in the red dotted-line box. Directions of genes and ORFs are indicated by arrows above the central thick line and the names are as given above. Green boxes represent truncated Δ*comM*, turquoise blue boxes represent insertion element, IS*Aba1*, and orange boxes represent genes conferring antibiotic resistance. Numbered boxes in gray represent genes encoding for hypothetical proteins. Comparison between structures of **(A)** AC12-RI1, **(B)** AC30-RI1, **(C)** AC29-RI1, **(D)** AbaR22, and **(E)** RI_MDR−TJ_, revealed similarities in the islands, as indicated by the same colors and red dotted-line box.

The genomes of *A. baumannii* AC29 and AC30 contained an AbaR4-type resistance island (RI) which interrupts the *comM* gene (Nigro and Hall, 2012). The island found in AC29 is 23 kb and designated AC29-RI1 whereas in AC30, it is 26 kb and designated AC30-RI1. Tn*6022* and/or ΔTn*6022* is part of the important features for this type of RI; in AC29-RI1, only the entire *tniC* gene is deleted in ΔTn*6022* whereas AC30-RI1 carries a complete copy of Tn*6022* (Figure 2). In AC29-RI1, the ΔTn*6022* is missing the *tniC* gene on its far left end, but full-length *tniD* and *tniE* genes are present in the transposon. AC30-RI1 contains the full length Tn*6022*. In both AC29-RI1 and AC30-RI1 islands, only one copy of either ΔTn*6022* or Tn*6022* is found.

Drug resistance genes found in the AC29-RI1 and ACRI30-1 islands are *sulI* (conferring sulphonamide resistance), *bla*_OXA−23_ (conferring carbapenem resistance), *tetA* and *tetB* (conferring tetracycline resistance), and *strA* with *strB* (conferring streptomycin resistance). The *bla*_OXA−23_ gene is flanked by two copies of the IS*Aba1* insertion element in a composite transposon structure similar to Tn*2006* (Figure 2). Tn*2006* comprises of the *bla*_OXA−23_ carbapenemase-encoding gene, a DEAD/DEAH box helicase-like gene and an ATPase gene (*yeeA*) flanked by two copies of IS*Aba1*.

### Plasmids of *A. baumannii* AC29 and AC30

#### A small 8.7 kb plasmid found in both AC29 and AC30

**Figure 3:**
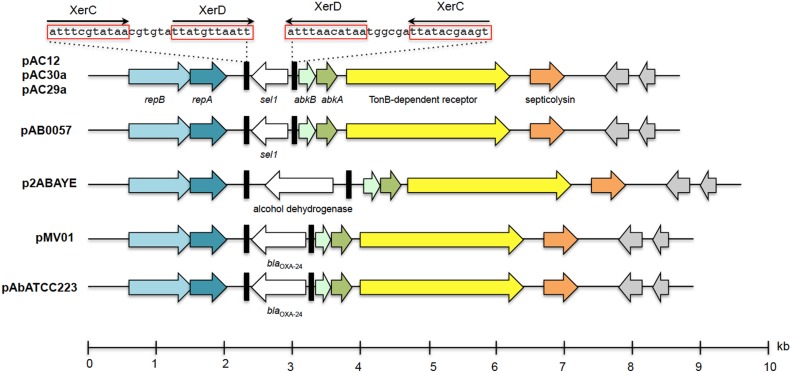
**Linear map showing the genetic organization of plasmids pAC29a, pAC30a, and pAC12 in comparison with other similar *A. baumanii* plasmids**. The XerC/XerD-like recombination sites are indicated in black rectangular boxes with the nucleotide sequences as indicated above the boxes. The two plasmid replication genes, *repB* and *repA*, are indicated in blue whereas the putative *abkA/abkB* toxin-antitoxin genes are indicated in green arrows. Hypothetical ORFs are shaded gray. The TonB-dependent receptor gene is indicated in yellow whereas the putative septicolysin gene is in orange.

Sequence analysis revealed the presence of a small 8.7 kb plasmid in both the genomes of *A. baumannii* AC29 and AC30, designated pAC29a and pAC30a, respectively (Table 1). These plasmids contained a total of nine coding sequences (CDS) (Figure 3). No antibiotic resistance gene was found on these plasmids. Two plasmid replication genes, designated *repA* and *repB*, were found on these plasmids along with an iteron sequence made up of four direct repeats of 5′-ATA TGT CCA CGT TTA CCT TGC A-3′ located 53 nucleotides upstream of the *repB* gene. Other features on this cryptic plasmid are a Sel1 repeat protein-encoding gene (sel1) which is flanked by XerC/XerD-like recombination sites in an inverted repeat formation, an outer membrane TonB-dependent receptor gene, a gene encoding for putative septicolysin and two hypothetical protein-encoding genes. A toxin-antitoxin (TA) system designated AbkB/AbkA (Mosqueda et al., 2014) was also encoded on these 8.7 kb plasmids.

#### PAC30b, a 16.2 kb resistance plasmid found in *A. baumannii* AC30

**Figure 4:**
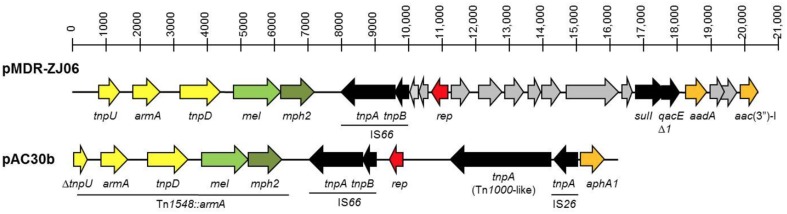
**Linear map comparison of the Tn*1548*::*armA* region in plasmid pAC30b from *A. baumannii* AC30 and pMDR-ZJ06 from *A. baumannii* MDR-ZJ06 (NC_017172)**. The Tn*1548*::*armA* region and insertion elements (IS) were underlined and indicated in the diagram.

Plasmid pAC30b is 16,236 bp in size and is found only in *A. baumannii* AC30. pAC30b contained 11 CDS and is a resistance plasmid, as indicated by the presence of the 16S rRNA methylase gene (*arm*A) and aminoglycoside 3′-phosphotransferase gene (*aph*A1) which confer resistance to aminoglycosides, along with macrolide 2′-phosphotransferase (*mph2*) and macrolide efflux protein-coding (*mel*) genes which confer resistance to macrolides (Figure 4; Zhou et al., 2011). The *armA, mph2*, and *mel* genes along with adjacent putative transposase genes *tnpD* and *tnpU* make up a small 7 kb RI designated Tn*1548*::*armA* which have been reported in several other plasmids from *Enterobacteriaceae* (Dolejska et al., 2013).

The *tnpU* putative transposase gene located at the 3′-end of Tn*1548::armA* is truncated in pAC30b whereby only the last 480 bp of the 836-bp gene could be found and was thus designated Δ*tnpU*. The pAC30b plasmid encodes a number of putative transposases: Δ*tnpU* and *tnpD* from Tn*1548::armA, tnpA*, and *tnpB* transposases from IS*66*, a 2888-bp *tnpA* from the Tn*3* family of transposases (or Tn*1000*-like) and immediately adjacent to that, another smaller 704-bp *tnpA* transposase belonging to IS*26*. The aminoglycoside resistance gene *aphA1* is found downstream from IS*26*.

#### A large ca. 70 kb conjugative plasmid in the genomes of *A. baumannii AC29* and AC30

**Figure 5:**
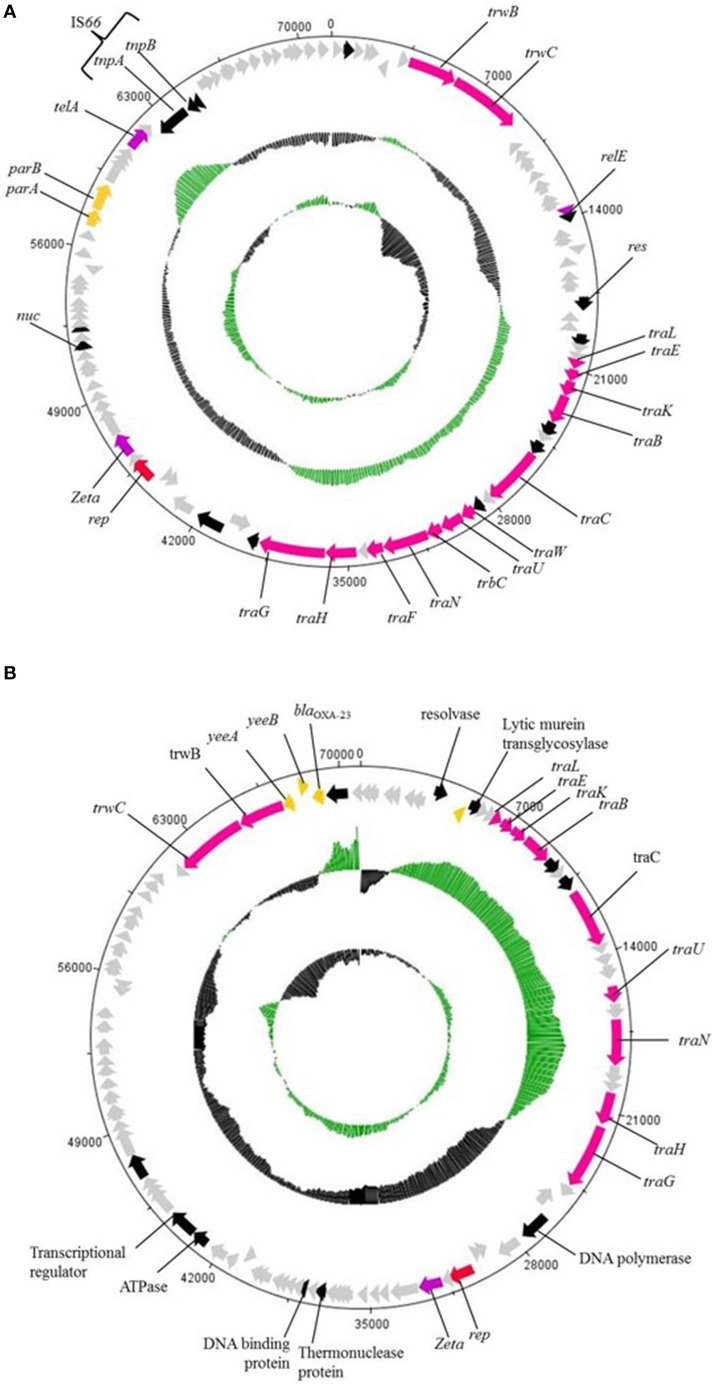
**Circular map of the ~70 kb plasmids pAC30c (A) and pAC29b (B)**. Outer circle of the map represents ORFs found in the plasmid, gray ORFs are genes encoding for hypothetical proteins. The two inner circles represent GC plot and GC skew, whereby the green circle stands for above average and black circle stands for below average G+C content. Red colored arrow represents plasmid replication gene, *rep*; orange colored arrow represents resistant determinants; pink colored arrow represents *tra* genes; black colored arrow represents genes encoding proteins with known functional homologs; purple colored arrow represents putative toxin gene homologs of toxin-antitoxin systems; and gray colored arrow represents genes encoding hypothetical proteins.

Both *A. baumannii* AC29 and AC30 were found to harbor similar conjugative plasmids of ca. 70 kb in size designated pAC29b and pAC30c, respectively. There are a total of 101 CDS in the 74,749 bp pAC29b whereas pAC30b is 71,433 bp and contains a total of 96 CDS. Both pAC29b and pAC30c encode a complete *tra* locus (Figure 5) similar to the *tra* locus found in pACICU2 (Iacono et al., 2008), p2ABTCDC0715 (Chen et al., 2011), pAb-G7-2 (Hamidian and Hall, 2014), and pA85-3 (Hamidian et al., 2014). Genes for the T4SS in pAC30c and pAC29b are clustered in two separate regions: the main *tra* region responsible for mating pair formation spans approximately 20 kb from *traL* to *traG*, and a second smaller region containing *traD* and *traI* (or *trwB* and *trwC*) responsible for plasmid mobilization. In pAC30c, the space between these two regions contains several hypothetical ORFs and a solitary *relE* toxin gene without the corresponding *relB* antitoxin gene (Figure 5).

Plasmid pAC29b is larger by 3316 bp when compared to pAC30c. However, there is a 14,374 bp fragment in pAC30c which has no homology with pAC29b. The additional fragment found in pAC29b included the β-lactamase gene *bla*_OXA−23_ and two transcriptional regulators, *tetR* and *asnC*. Other identifiable genes within this pAC29b-unique fragment include *yeeA* (encoding DNA methyltransferase), *yeeB* (encoding ATP-dependent helicase), *yhbS* (encoding N-acetyltransfease), *mscM* (encoding mini-conductance mechanosensitive channel), *iutA* (encoding ferric aerobactin receptor), *lrp* (encoding leucine-responsive regulatory protein), and *aroQ* (encoding monofunctional chorismate mutase). Thus, AC29 harbors two copies of *bla*_OXA−23_—one within the AbaR4-like AC29RI-1 island in the chromosome, and another encoded on the pAC29b plasmid. However, plasmid pAC30c does not encode for any known resistance determinant.

### Investigations into the possible contributors of polymyxin resistance in *A. baumannii* AC30

**Figure 6:**
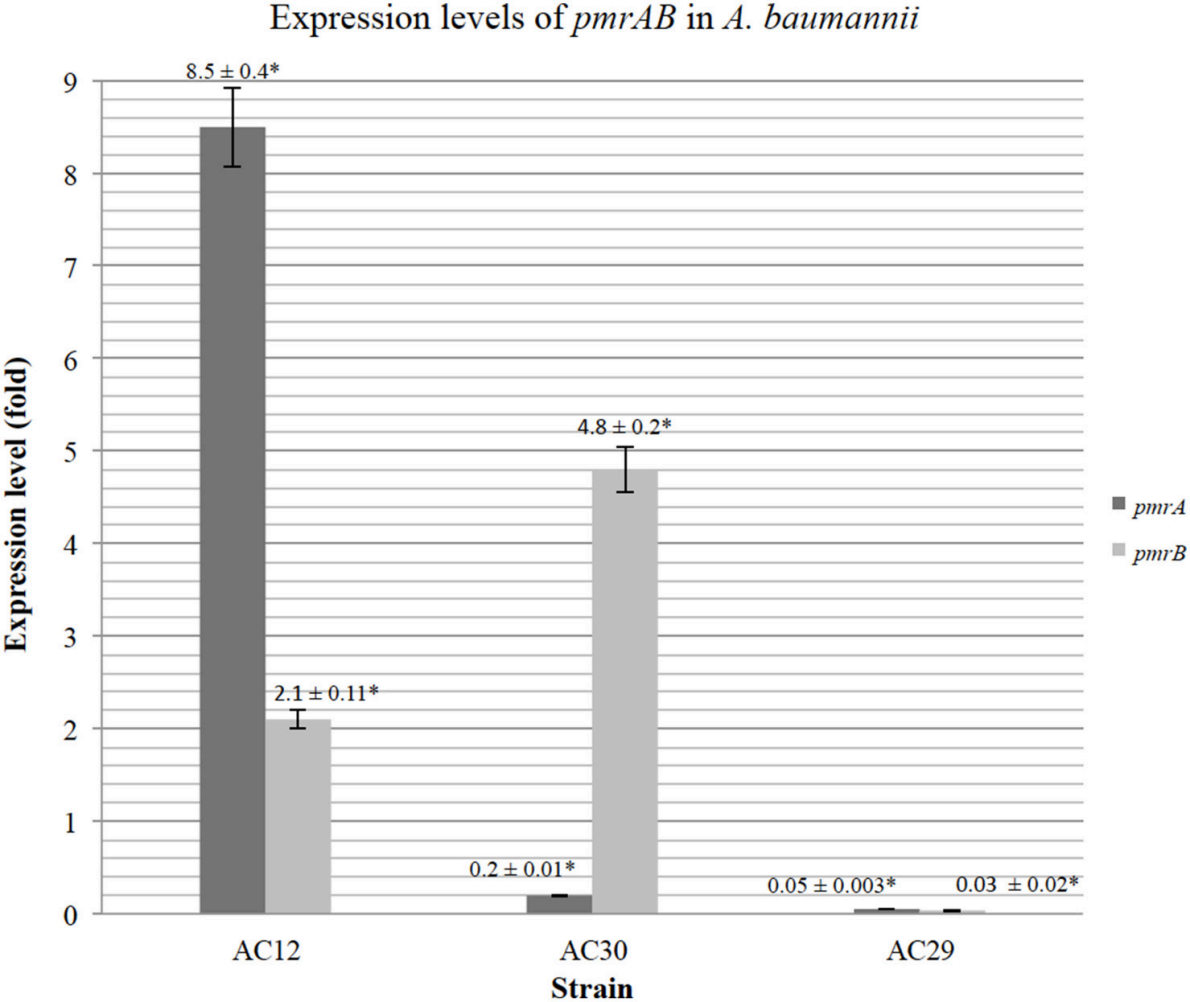
**Relative expression levels of *pmrAB* of the polymyxin-resistant *A. baumannii* AC12 and AC30 as compared to the polymyxin-susceptible *A. baumannii* AC29 as determined by Quantitative Real-Time reverse transcriptase-PCR (qRT-PCR)**. The data represents the mean fold change ± standard deviation (SD; indicated as error bars in the graph) taken from three replicates performed for each qRT-PCR reaction. Asterisk (^*^) indicate statistical significance, as determined by using two-tailed, unpaired Student's *t*-test with *p* < 0.05.

To investigate if differential expression of *pmrAB* occurred in the polymyxin-resistant AC30 as well as AC12 strains, relative quantification of the *pmrAB* transcript levels were determined by qRT-PCR with *A. baumannii* ATCC19606 as the polymyxin-susceptible control. When compared to ATCC19606, the relative expression levels of *pmrA* in AC30 was surprisingly lower at about 0.2-folds but levels of *pmrB* were higher at 4.8-folds (Figure 6). In contrast, the other polymyxin-resistant strain, AC12 displayed about 8.5-folds higher *pmrA* expression levels and about two-folds higher *pmrB* levels in comparison with ATCC19606. The polymyxin-susceptible AC29 showed dramatically lower *pmrAB* expression, at about 0.05- and 0.03-folds, respectively.

**Figure 7:**
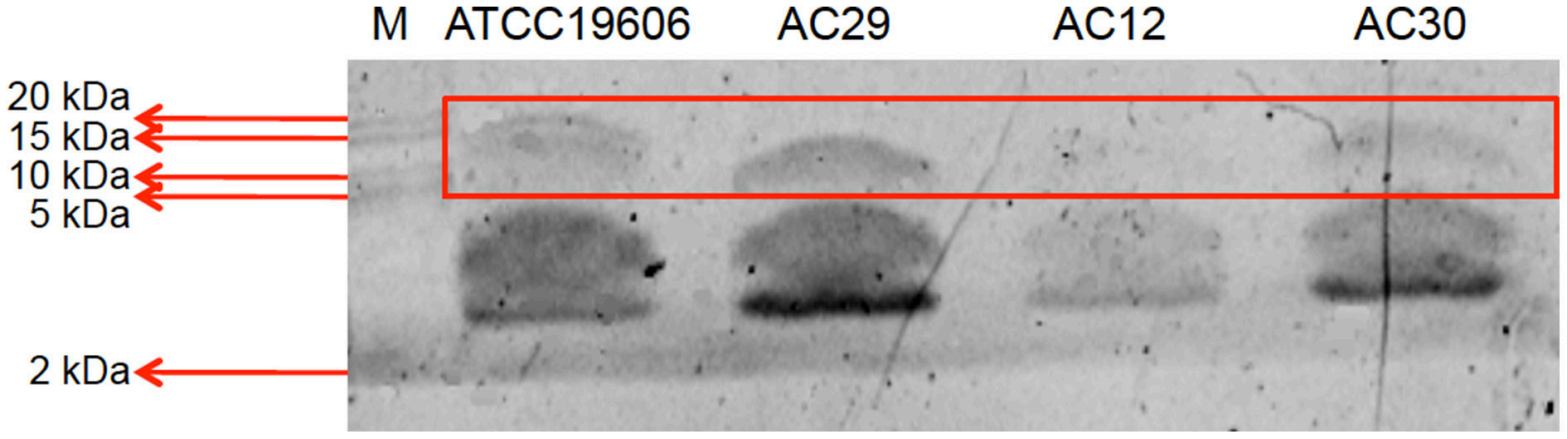
**SDS-PAGE analysis of the extracted lipopolysaccharide (LPS) layer of the *A. baumannii* ATCC19606 (control; polymyxin susceptible strain), AC12 (polymyxin resistant strain), AC29 (polymyxin susceptible strain), and AC30 (polymyxin resistant strain)**. M stands for the protein marker Precision Plus Protein Dual Xtra Standards (BioRad) with the sizes as indicated in kDa.

LPS from the two polymyxin-resistant strains, AC12 and AC30, along with two polymxyin-susceptible strains AC29 and ATCC19606 were extracted and analyzed on 15% SDS-polyacrylamide gels (Figure 7). SDS-PAGE of the extracted LPS yielded a band of ~10 kD, which was within the expected molecular weight (between 6 and 10 kDa) for LPS. Results indicated that the LPS in the polymyxin resistant strains AC12 and AC30 were not totally absent as had been previously reported in other polymyxin-resistant strains (Moffatt et al., 2010, 2011; Hood et al., 2013) but the intensity of the LPS band was considerably less when compared with the LPS band of the polymyxin-susceptible strains.

### AmpC-mediated resistance to extended-spectrum cephalosporins

The AmpC cephalosporinase encoded by *A. baumannii* AC29 and AC30 displayed identical non-synonymous mutations, R80S and G246S (with reference to ADC-7, the reference *A. baumannii*-encoded AmpC; Hujer et al., 2005; Rodríguez-Martínez et al., 2010) to that found in *A. baumannii* AC12 (Lean et al., 2015) but the effects of these mutations on cephalosporin resistance have not been reported. To investigate if the mutations in the *bla*_AmpC_ gene harbored by *A. baumannii* AC29 and AC30 has any effect on resistance against β-lactams especially cephalosporins, these genes were cloned and expressed in *E. coli* BL21 through the IPTG-inducible T7 promoter of the pET30a vector. Recombinant *E. coli* BL21 carrying the *bla*_AmpC_ from AC29 and AC30 displayed resistance to ceftazidime, cefepime, aztreonam, and even imipenem (Table 2). Thus, the *bla*_AmpC_ encoded by AC29 and AC30 is likely an extended-spectrum *Acinetobacter*-derived AmpC (ESAC).

**Table 2:** **MIC values of the *ampC* clones in IPTG-induced *E. coli* BL21 toward selected antibiotics**.

**Clones**	**MIC value (μg/mL)**			
	**Aztreonam**	**Ceftazidime**	**Imipenem**	**Cefepime**
BL21_ampC-AC12	16	16	32	16
BL21_ampC-AC30	16	16	32	16
BL21_ampC-AC29	16	16	32	16
Control (BL21_pET30a)	2	2	2	2

### Other resistance determinants and efflux pumps

Besides the *bla*_AmpC_-encoded cephalosporinase (belonging to Ambler Class C β-lactamases) and the Class D carbapenemases encoded by *bla*_OXA−23_ and *bla*_OXA−51_ that were described in the previous sections, two other β-lactamase genes were found in the genomes of AC29 and AC30 (Table 3). These are the *bla*_TEM_ gene which encodes class A extended-spectrum β-lactamase and a gene identified as belonging to the metalo-β-lactamase family (Ambler Class B). The contributions of these genes to the β-lactam resistance phenotype of AC12 and AC30 are currently unknown.

**Table 3:** **Genes conferring antibiotic resistance found in *A. baumannii* AC29 and AC30**.

**Antibiotics**	**Genes**	**Contig location**		**Products**
		**AC29**	**AC30**	
Aminoglycosides	*aad*A	3_40	10_40	Adenyltransferase
	*aad*A1a	3_40	10_40	Aminoglycoside adenylyltransferase
	*aph*A1b	88_1	91_1	Aminoglycoside 3′-phosphotransferase
	*str*A	54_7	38_7	Phosphotransferase
	*str*B	54_6	38_6	Phosphotransferase
Beta-lactams	*amp*C	14_1	37_1	Beta-lactamase Class C
	*bla*_TEM_	85_1	87_1	Beta-lactamase TEM
	Class A beta-lactamase	34_58	6_58	Beta-lactamase Class A
	MBL	16_33	3_184	Metallo-beta-lactamase family protein
	*bla*_OXA−23_	60_16	40_16	Beta-lactamase OXA-23
	*bla*_OXA−51_	53_6	60_1	Beta-lactamase OXA-51
Carbapenems	*car*O	16_123	6_123	Putative porin protein associated with imipenem resistance
Chloramphenicol	*cml*A	35_12	47_5	Major facilitator superfamily permease
	*cml*A	35_12	47_5	cmlA transporter
Fluoroquinolones	*par*C	45_19	49_19	DNA topoisomerase IV subunit A
	*parE*	17_100	1_100	DNA topoisomerase IV subunit B
	*gyr*A	16_68	3_149	DNA gyrase subunit A
	*gyr*B	40_38	23_38	DNA gyrase subunit B
Tetracyclines	*tet*A, Class A	54_1	38_1	Tetracycline resistance protein
	*tet*R	54_2	38_2	Tetracycline repressor protein
Trimethoprim	*dhfr*I	33_27	5_27	Dihydrofolate reductase
Sulfonamides	*sul*I	16_16	57_14	Dihydropteroate synthase
Polymyxin B	*pmr*A	15_53	34_55	Polymyxin resistance component, PmrA
	*pmr*B	15_54	34_54	Polymyxin resistance component, PmrB
	*pmr*C	15_55	43_1/39_1	Polymyxin resistance component, PmrC
Colistin	*lpx*A	29_1	13_136	UDP-N-acetylglucosamine acyltransferase
	*lpx*B	38_1	8_113	Lipid-A-disaccharide synthase
	*lpx*C	12_51	1_129	UDP-3-O-[3-hydroxymyristoyl] N-acetylglucosamine deacetylase
	*lpx*D	19_75	13_138	UDP-3-O-[3-hydroxymyristoyl] glucosamine N-acyltransferase
	*lpx*H	17_129	35_87	UDP-2,3-diacylglucosamine hydrolase
	*lpx*K	12_53	8_19	Tetraacyldisaccharide 4′-kinase

One of the main mechanisms of fluoroquinolone resistance is mutations that alter the drug targets. The targets of fluoroquinolone action are the bacterial enzymes DNA gyrase (encoded by *gyrA* and *gyrB*) and DNA topoisomerase IV (encoded by *parC* and *parE*), both of which work together in replication, transcription, recombination and repair of DNA (Jacoby, 2009). The AC29 and AC30-encoded *gyrA* and *parC* showed the Ser → Leu amino acid substitutions at positions 83 and 80, respectively, which have been implicated in fluoroquinolone resistance (Wisplinghoff et al., 2003; Fournier et al., 2006; Maragakis and Perl, 2008). However, four additional novel point mutations (G145D, S118G, L644P, and T872A) were observed in the AC29 and AC30-encoded *gyrA*. Whether these mutations contribute to fluoroquinolone resistance in *A. baumannii* AC29 and AC30 would require further investigations.

Multidrug efflux pumps and porins also play important roles in *A. baumannii* antimicrobial resistance (Vila et al., 2007). Among the five major families of bacterial efflux pumps (i.e., RND, MFS, APC, ABC, and MATE; Table 4), the RND family is widely disseminated in Gram-negative bacteria (Poole, 2004; Bonomo and Szabo, 2006; Nordmann and Poirel, 2008; Wieczorek et al., 2008). The genome analysis of *A. baumannii* AC29 and AC30 showed the presence of the complete *adeABC* genes along with its two-component regulatory genes, *adeRS*. Another member of the RND family encoded by *adeIJK* was also present in AC29 and AC30. However, a third RND efflux pump encoded by the *adeFGH* operon was absent in both isolates. Overexpression of *adeABC* and to a certain extent, *adeIJK*, has also been associated with the multidrug resistance phenotype in *A. baumannii* (Vila et al., 2007) and it would be of interest to investigate if this is likewise in *A. baumannii* AC29 and AC30.

**Table 4:** **Drug transporter and efflux pumps found in *A. baumannii* AC29 and AC30**.

**Drug transporters**	**Genes**	**Contig location**		**Gene products**
		**AC29**	**AC30**	
APC family transporter	*cyc*A	16_60	3_157	D-serine/D-alanine/glycine transporter
	*cyc*A2	3_70	10_70	amino acid APC transporter
		3_71	10_71	amino acid APC transporter
	*mmu*P	67_1	22_32	S-methylmethionine APC transporter
	*pro*Y	17_59	1_59	proline-specific permease ProY
RND family transporter	*ade*A	31_11	16_59	membrane-fusion protein
	*ade*B	31_10	16_60	cation/multidrug efflux pump
	*ade*C	31_9	16_61	multidrug efflux protein AdeC
	*ade*I	15_38	34_39	multidrug efflux protein AdeI
	*ade*J	15_39	34_40	multidrug efflux protein AdeJ
	*ade*K	15_40	34_41	multidrug efflux protein AdeK
	*ade*R	31_21	16_58	AdeR
	*ade*S	31_13	16_57	AdeS
	*fus*E	9_83	19_57	Putative FusE-MFP/HlyD membrane fusion protein
	*mdt*A	20_59	14_55	multidrug ABC transporter
	*nol*F	35_29	4_13	NolF secretion protein
	RND efflux pumps	35_29	4_13	NolF secretion protein
MFS family transporter	*emr*B	26_33	27_212	major facilitator superfamily multidrug resistance protein
	*fsr*	25_59	27_36	major facilitator superfamily permease
	*bcr*	35_12	47_5	MFS superfamily bicyclomycin/multidrug transport protein
	MFS superfamily protein	12_101	13_186	transporter, major facilitator family protein
	MFS transporter	19_98	8_90	major facilitator superfamily permease
	*yga*Y	8_35	18_33	transporter, major facilitator family protein
	*nor*M	9_13	19_5	multidrug ABC transporter
	*cmr*	22_155	22_42	major facilitator superfamily multidrug/chloramphenicol efflux transporter
ABC family transporter	ABC efflux pump	9_13	19_5	multidrug ABC transporter
Na+ driven transporters	Na+ driven efflux pump	17_88	1_88	Na+-driven multidrug efflux pump
Efflux pumps	*abe*M	9_13	19_5	multidrug efflux pump AbeM
	*dmt*	16_81	3_136	EamA-like transporter family protein
	*ywf*M	56_27	12_128	DMT family permease
	MATE efflux pump	17_88	1_88	MATE efflux family protein
SMR	*smr*	22_155	14_62	multidrug resistance protein, SMR family
		20_66	14_1	Smr protein/MutS2

## Discussions

### Whole genome sequencing and sequence types

In this study, we applied comparative genomics on two *A. baumannii* strains that are indistinguishable by PFGE but have different antibiograms to better understand their mechanism of resistance. Although these two strains only differed by their susceptibility toward polymyxin B (AC29 was susceptible whereas AC30 was resistant), detailed genomic analysis indicated a number of differences in the RI structures and the plasmid contents. Both *A. baumannii* AC29 and AC30 were typed as ST195 and, not surprisingly, their closest neighbor was *A. baumannii* AC12 which was also another ST195 strain as previously reported from (Lean et al., 2015). Another close neighbor was *A. baumannii* M1, an isolate from Malaysia that was recently deposited and from its genome sequence, was deduced to be also ST195. All these strains were clearly clustered in the IC-II group of global *A. baumannii* clones (Figure 1). Strains that were typed as ST195 under the Bartual MLST scheme are grouped into the clonal complex 92 (CC92) which are characterized as under IC-II (Kim et al., 2013; Zarrilli et al., 2013).

### The AbaR4-type resistance island

One of the known hotspots for the insertion of resistance islands in *A. baumannii* is the *comM* gene (Post et al., 2010; Zhou et al., 2011; Huang et al., 2012; Kim et al., 2013). The genomes of *A. baumannii* AC29 and AC30 also contained a similar island within *comM* but with slight differences. Previous analysis of the *A. baumannii* AC12 genome showed the presence of a 23 kb AbaR4-type island designated AC12-RI1 within *comM* (Lean et al., 2015) which consisted of a backbone of Tn*6167* and a truncated version of Tn*6022* designated ΔTn*6022*. In all three islands (i.e., AC12RI-1, AC29RI-1, and AC30RI-1), only one copy of either ΔTn*6022* or Tn*6022* is found whereas other similar resistance islands usually contained two copies of ΔTn*6022* and/or Tn*6022*. For instance, in RI_MDR−TJ_ and AbaR22, another copy of the complete Tn*6022* was located next to the *tetA*(B) gene (Figure 2). Besides that, the *bla*_OXA−23_ gene in AC12-RI1, AC29-RI1, and AC30-RI1 was found flanked by two copies of the IS*Aba1* insertion element in a composite transposon structure similar to Tn*2006* (Figure 2). The presence of IS*Aba1* upstream of *bla*_OXA−23_ may contribute to carbapenem resistance by increasing the expression level of the gene by virtue of its outward-directed promoter (Zhu et al., 2013). The Tn*2006*-like structure was inserted immediately downstream from ΔTn*6022* or Tn*6022*, and interestingly in AC12-RI1 and AC29-RI1, the *orf4* gene found at the right-most end of ΔTn*6022* was duplicated, resulting in the ΔTn*2006*-like structure being flanked by *orf4* on either end (Figure 2). Such an arrangement has not been reported in similar resistance islands before. Besides that, comparison between the Tn*2006* present in the three RIs and Tn*6167* showed some differences. Tn*6167* harbors Tn*2006*, but at a different location whereas both RI_MDR−TJ_ and AbaR22 do not contain Tn*2006*. However, the right end of the islands were identical with Tn*6167*, RI_MDR−TJ_ and AbaR22 and comprises of *tetA*(B)-*tetR*(B), the small mobile element CR2, *strA*-*strB* and *orf4b*, a hypothetical ORF related to *orf4* of Tn*6022* and Tn*6022*Δ1 (Figure 2).

The structures of AC29-RI1 and AC30-RI1 indicated that they are novel variants of the AbaR4-type RIs that were recently reported in a survey of RIs found in *A. baumannii* strains throughout Asia (Kim et al., 2013). Kim et al. (2013) also reported that AbaR4-type RIs were commonly found among carbapenem-resistant CC92 strains which included the ST195 lineage.

### Plasmids of *A. baumannii* AC29 and AC30

#### A small 8.7 kb plasmid found in both AC29 and AC30

Plasmid pAC29a and pAC30a were almost identical to pAC12 (Lean et al., 2015), pAB0057 (Adams et al., 2008), p1ABTCDC0715 (Chen et al., 2011), and pPKAB07 (Saranathan et al., 2014). A similar *A. baumannii* plasmid, pABVA01 (8963 bp), was found to harbor the *bla*_OXA−24_ carbapenese gene flanked by XerC/XerD-like recombination sites (D'Andrea et al., 2009) and this arrangement was also subsequently reported in the 8771 bp plasmid pMMCU1 (Merino et al., 2010). The XerC/XerD-like recombination sites were present in pAC29a/pAC30a but with the *selI* gene instead of *bla*_OXA−24_ within the potential recombination region. The pAC12 plasmid from *A. baumannii* AC12 (Lean et al., 2015) and pAB0057 from *A. baumannii* AB0057 (Adams et al., 2008) also contained the *sel1* gene in between the XerC/XerD sites. The function of the *sel1* gene is currently unknown. Other similar *Acinetobacter* plasmids harbored different sized fragments between the XerC/XerD recombination sites with p2ABAYE from *A. baumannii* AYE (Fournier et al., 2006) for example, harboring a putative alcohol dehydrogenase gene (Figure 3). The *bla*_OXA−24_ gene is so far reported only in strains from Italy and Spain (D'Andrea et al., 2009; Merino et al., 2010). This was corroborated in a recent multicenter study which showed that the *bla*_OXA−24_/*bla*_OXA40−like_ genes in *A. baumannii* strains isolated from Spain was predominantly carried by small 8–12 kb plasmids with two of the sequenced plasmids, pAbATCC223 and pAbATCC329, harboring the *bla*_OXA−24_ gene in between the XerC/XerD sites (Mosqueda et al., 2013). Xer recombination is a site-specific recombination mechanism involved in events such as the integration of phage CTX-Φ at the *dif1* site in the *Vibrio cholerae* chromosome (D'Andrea et al., 2009). Moreover, the proteins required for Xer recombination, such as the XerC and XerD recombinases and PepA are reportedly encoded in the chromosome of several *A. baumannii* strains (Merino et al., 2010). Thus, the XerC/XerD-like sites on these 8.7 kb plasmids could act as site-specific recombination targets responsible for mobilization of discrete gene modules such as *bla*_OXA−24_ and *sel1* within *Acinetobacter* plasmids (D'Andrea et al., 2009; Merino et al., 2010).

Other highlights of this small plasmid are the presence of a toxin-antitoxin (TA) system designated AbkB/AbkA (Mosqueda et al., 2014) and a gene encoding a possible TonB-dependent receptor protein. Most TA systems are usually characterized by two co-transcribed genes with the antitoxin gene preceding the toxin gene (Chan et al., 2012; Hayes and Kêdzierska, 2014). AbkB/AbkA seems to differ from canonical TA systems as the *abkB* toxin gene precedes the *abkA* antitoxin gene. To date, only three characterized TA loci have been reported to display this unusual genetic arrangement: the *mqsRA* (Brown et al., 2009), the *higBA* (Tian et al., 1996), and *hicAB* (Jørgensen et al., 2009) modules. AbkB/AbkA was previously identified as one of the four functional TA systems in *A. baumannii* (it was designated SplT/SplA or DUF497/COG3914) whereby overexpression of the toxin was shown to inhibit growth in *E. coli* and this was overcame by co-expression of the cognate antitoxin (Jurenaite et al., 2013). The AbkB (or SplT) toxin was shown to inhibit translation when overexpressed in *E. coli* with cleavage of *lpp* mRNA and transfer-messenger RNA (tmRNA) demonstrated, thus indicating that the AbkB toxin likely functions as an endoribonuclease or RNA interferase. The AbkB/AbkA locus was found to be highly prevalent in small plasmids of *A. baumannii* clinical strains (88.6% prevalence among 476 clinical isolates from Lithuania; Jurenaite et al., 2013). The presence of a TA system on these plasmids would explain their stability in the absence of any apparent selection pressure, particularly for the small plasmids without the *bla*_OXA−24_/*bla*_OXA40−like_ gene such as pAC30a and pAC29a.

Some TonB-dependent receptors, in particular BauA, play important roles in the acquisition of iron in *A. baumannii* (Dorsey et al., 2004; Mihara et al., 2004) with recent transcriptomic and proteomic analyses indicating approximately 20 TonB-dependent receptors in *A. baumannii*, some of which are regulated by iron (Antunes et al., 2011; Eijkelkamp et al., 2011; Nwugo et al., 2011). TonB-dependent receptors are usually found in the outer membrane where they interact with TonB and associated inner membrane proteins (ExbB and ExbD) that provide energy needed to transport host iron-carrier and iron-siderophore complexes into the periplasm once these complexes are bound to the TonB-dependent receptors (Zimbler et al., 2013). Thus, along with TonB, the TonB-depended receptors play an important role in the virulence of *A. baumannii*. Whether the TonB-dependent receptor protein encoded by these small plasmids plays a similar role in iron acquisition and hence, virulence, awaits further experimentation. Another possible virulence-associated gene encoded in these small plasmids is found downstream of the gene encoding the TonB-dependent receptor. This gene is predicted to encode a 152-amino acids-residue protein homologous to septicolysin, a putative virulence factor (Mosqueda et al., 2014). Septicolysin is a member of thiol-activated cytolysins which have been implicated in the pathogenesis of infections by several Gram-positive pathogens such as *Clostridium perfringens, Listeria monocytogenes*, and *Streptococcus pneumoniae* and are characterized by their cytolytic activity for eukaryotic cells (Billington et al., 2000). With two putative virulence factors encoded on these small plasmids, it would therefore be of interest to investigate if these plasmids play a role in the virulence and pathogenesis of *A. baumannii* infections, especially in view of their prevalence among clinical *A. baumannii* isolates.

#### pAC30b, a 16.2 kb resistance plasmid found in *A. baumannii* AC30

Both pAC30b and pMDR-ZJ06 are resistance plasmids, as indicated by the presence of genes which confer resistance to aminoglycosides and macrolides within the 7 kb Tn*1548::armA* resistance island (Zhou et al., 2011). Interestingly, Tn*1548::armA* is located in the chromosome of *A. baumannii* AC12 (Lean et al., 2015) as well as in *A. baumannii* AC29. In both cases, Tn*1548*::*armA* was found downstream of a cluster of five genes encoding proteins annotated as paraquat-inducible protein A and protein B. The pMDR-ZJ06 plasmid harbors a class 1 integron encoding the aminoglycoside acetyltransferase (*aacC1*) and adenyltransferase (*aadA1*) along with *sulI* that confers sulphonamide resistance (Zhou et al., 2011) but this integron was absent in pAC30b.

Besides pMDR-ZJ06, pAC30b also shared sequence identity with p3BJAB0868 from *A. baumannii* BJAB0868 and p2BJAB07104 from *A. baumannii* BJAB07104 (Zhu et al., 2013) and this is mainly an approximately 10 kb fragment that spanned Tn*1548*::*armA*, IS*66* and the *rep* gene. Two other parts of pAC30b that were identical with pMDR-ZJ06, p3BJAB0868, and p2BJAB07104 are IS*26* and the *aphA1* gene, both of which were in different locations in pAC30b when compared with pMDR-ZJ06 (Figure 4), p3BJAB0868, and p2BJAB07104. The *aphA1* gene is flanked by two copies of IS*26* in a composite transposon-like structure designated Tn*6210* in p3BJAB0868 and p2BJAB07104 (Zhu et al., 2013) as well as pMDR-ZJ06 but in pAC30b, only one copy of IS*26* is found adjacent to *aphA1*. It is possible that a deletion had occurred in pAC30b that took out the other copy of IS*26* as well as part of the *tnpU* gene of Tn*1548*::*armA* which is located adjacent to the IS*26*-*aphA1* structure. All three plasmids pMDR-ZJ06, p3BJAB0868, and p2BJAB07104 which were isolated from *A. baumannii* strains in China, harbored a class 1 integron but this was absent in pAC30b.

It is noteworthy that in contrast to pAC30a and pAC29a, pAC30b has very few similar plasmids in other *A. baumannii* isolates besides pMDR-ZJ06, p3BJAB0868, and p2BJAB07104, suggesting that these plasmids are not so prevalent. What the four *A. baumannii* plasmids (namely, pAC30b, pMDR-ZJ06, p3BJAB0868, and p2BJAB07104) have in common are (1) a number of transposases encoded by IS elements and transposons, and (2) a lack of any plasmid stability genes such as toxin-antitoxin systems. Thus, there is a likelihood that these plasmids are much less stable as compared to the smaller pAC29a/pAC30a plasmid which is endowed with the AbkB/AbkA toxin-antitoxin system. Hence, in *A. baumannii* AC29 (and AC12), portions of pAC30b are chromosomally-located and there is no widespread occurrence of pAC30b-like plasmids in other *A. baumannii* strains.

#### A large ca. 70 kb conjugative plasmid in the genomes of *A. baumannii* AC29 and AC30

The *tra* locus in pAC29b and pAC30c encodes for a type IV secretion system (T4SS) which is related to the bacterial conjugation machinery and mediates horizontal gene transfer (Juhas et al., 2008). Plasmids pAb-G7-2, pA85-3, and even pACICU2 have been demonstrated to be transmissible by conjugation to other *A. baumannii* strains (Hamidian and Hall, 2014; Hamidian et al., 2014). It is thus very likely that pAC29b and pCA30c are also conjugative as the *tra* locus in these plasmids is almost identical. In pAb-G7-2, the space between these two regions contained the aminoglycoside resistance transposon, Tn*aphA6* (Hamidian and Hall, 2014) whereas in pA85-3, the AbaR4 resistance island is located in the same area (Hamidian et al., 2014). In pAC30c, this region contained several hypothetical ORFs and a solitary *relE* toxin gene without the corresponding *relB* antitoxin gene. However, the two genes flanking *relE* could perhaps function as the antitoxin as they encode for hypothetical proteins of about the same size as the putative RelE toxin. Toxin-antitoxin pairs are usually about the same size with a few exceptions such as the Zeta toxin which is much larger (~270 amino acids) as compared to their cognate Epsilon antitoxin (~90 amino acids; Chan et al., 2012; Jurenaite et al., 2013). Although toxins usually interact with their cognate antitoxin pair, sometimes mixing and matching between different toxin and antitoxin families do occur (Chan et al., 2012; Hayes and Kêdzierska, 2014). Nevertheless, the functionality of the plasmid pAC30c and pAC29b-encoded *relE* toxin gene needs to be ascertained.

Both pAC30c and pAC29b carry the *rep* gene designated *rep*Aci6, similar to pACICU2, pAb-G7-2, and pA85-3. Another solitary toxin, this time from the Zeta family, is located downstream from the *rep*Aci6 gene in both plasmids. Experimental evidence had suggested that its overexpression is non-toxic to *E. coli* and thus, may not function as a typical toxin (Jurenaite et al., 2013). Its putative antitoxin partner, located upstream of its reading frame, does not bear any homology to the Epsilon antitoxin. These solitary Zeta-like toxins have been observed in several other plasmids in diverse bacterial species and their function is currently unknown (Chan et al., 2012; Jurenaite et al., 2013). In the absence of typical canonical toxin-antitoxin systems, the only other identifiable genes that could contribute to plasmid stability in pAC30c and pAC29b are the *parAB* genes which likely encode proteins that are involved in plasmid partitioning.

Plasmid pAC29b harbors a *bla*_OXA−23_ gene but unlike the *A. baumannii* A85 plasmid pA85-3 (Hamidian et al., 2014), the gene is not located within an AbaR4 island. Both pAb-G7-2 and pACICU2 harbor the aminoglycoside resistance gene *aphA6* within a composite transposon designated Tn*aphA6* (Hamidian and Hall, 2014; Hamidian et al., 2014). Plasmid pAC30c however, does not encode any known resistance determinant.

### Investigations into the possible contributors of polymyxin resistance in *A. baumannii* AC30

The mechanism for polymyxin resistance in *A. baumannii* has only recently been elucidated and the main mechanism appeared to be either covalent modification of the lipid A portion of LPS (Arroyo et al., 2011; Beceiro et al., 2011) or disruption of LPS biosynthesis (Moffatt et al., 2010; Park et al., 2011). By modification and/or mutations in the amino acid sequences, negative charges on the outer membrane can be reduced, leading to reduction in the affinity of the positively-charged polymyxin component, hence giving rise to polymyxin resistance (Adams et al., 2009; Arroyo et al., 2011; Beceiro et al., 2011). Overexpression of the two-component signal transduction system *pmrAB* and mutations within these genes, especially *pmrB*, were reported to contribute to polymyxin resistance (Adams et al., 2009; Arroyo et al., 2011; Park et al., 2011). These two genes are part of an operon along with *pmrC*, which encodes the enzyme responsible for the covalent addition of phosphoethanolamine to lipid A. Sequence analysis of *pmrCAB* from the polymyxin-resistant AC30 in comparison with the polymyxin-susceptible AC29 and other susceptible strains in the database (including ATCC19606 and ATCC17978) showed a P102H mutation within *pmrA*. Identical mutations were found within *pmrA* of AC12 (Lean et al., 2015) and other polymyxin-resistant isolates from Terengganu (Lean et al., 2014). Polymyxin-resistant isolate AC12 displayed higher *pmrA* expression levels (8.5-folds) and also higher *pmrB* levels (about two-folds) in comparison with ATCC19606. In contrast, the polymyxin-susceptible AC29 isolate showed dramatically lower *pmrAB* expression (0.05- and 0.03 folds). It should be noted that in this case, no isogenic polymyxin-susceptible strains for the polymyxin-resistant strains were available for comparison. Thus, the expression levels for AC29, AC30, and AC12 were compared with the non-isogenic reference strain ATCC19606 and therefore may not yield an accurate picture for the *pmrAB* levels. Nevertheless, the results do indicate upregulation particularly for *pmrB* in both the polymyxin-resistant isolates AC30 and AC12.

The *lpxA, lpxC*, and *lpxD* genes encode the first three enzymes in the lipid A biosynthesis pathway (Moffatt et al., 2010, 2011). No mutation was found in *lpxA*. In contrast, *lpxD* showed three amino acid mutations (S102T, V141I, R173G) in AC30 while *lpxC* had a K141R substitution. The *lpsB* gene encodes a glycosyltransferase is involved in the biosynthesis of the LPS core and was recently implicated in *A. baumannii* colistin resistance (Hood et al., 2013). Comparison of *lpsB* sequences indicated a H181Y substitution in AC30. Identical mutations in *lpxD, lpxC*, and *lpsB* were reported in the polymyxin-resistant *A. baumannii* AC12 (Lean et al., 2015); likewise, these and other mutations had been previously reported in the polymyxin-resistant *A. baumannii* strains from Terengganu (Lean et al., 2014). Results from the LPS analysis indicated that the LPS in the polymyxin resistant strains AC12 and AC30 were considerably less when compared with the LPS band of the polymyxin-susceptible strains, which has been previously reported in other polymyxin-resistant strains (Moffatt et al., 2010, 2011; Hood et al., 2013). This suggested that the mutations found in the *lpxD, lpxC*, and *lpsB* genes in the two polymyxin-resistant strains may have led to impairment but not a total loss of the LPS. It is likely that in the case of AC12 and AC30, polymyxin resistance could be the result of a combination of increased *pmrAB* expression leading to covalent modification of the lipid A moiety of LPS and possibly impaired LPS synthesis as well. Nevertheless, it should be noted that in a recent study (Hood et al., 2013), screening of transposon mutant libraries led to the identification of more than 20 genes that may be involved in inducible colistin resistance in *A. baumannii*. Most of these genes converged on pathways involved in osmotolerance, cell envelope biosynthesis along with protein folding (Hood et al., 2013). The role that these factors may play in the development of polymyxin resistance in the Terengganu *A. baumannii* strains would also need to be investigated.

### AmpC-mediated resistance to extended-spectrum cephalosporins

Resistance to β-lactam antibiotics via synthesis of β-lactamase encoded by the chromosome and/or plasmids is the most common resistance mechanism observed in *A. baumannii* (Bou and Martínez-Beltrán, 2000). Resistance to broad-spectrum cephalosporins in *A. baumannii* are usually related to the over production of extended spectrum β-lactamases (ESBL), especially AmpC-type β-lactamases designated ADCs (*Acinetobacter*-derived cephalosporinases) (Rodríguez-Martínez et al., 2010). ADCs typically hydrolyze penicillins, narrow- and extended-spectrum cephalosporins but not zwitterionic cephalosporins such as cefepime or carbapenems (Rodríguez-Martínez et al., 2010). However, extended-spectrum AmpCs (ESACs) have been reported in *A. baumannii* that confer reduced susceptibility to all cephalosporins and this includes ADC-33 (Rodríguez-Martínez et al., 2010) and ADC-56 (Tian et al., 2011).

Since the recombinant *E. coli* BL21 carrying the *bla*_AmpC_ from AC29 and AC30 displayed resistance to ceftazidime, cefepime, aztreonam, and even imipenem, this strongly suggests that the AmpC from AC29 and AC30 is an ESAC cephalosphorinase. ADC-33 possessed a P210R substitution together with a duplication of the Ala residue at position 215 within the Ω loop, both of which are required for extended spectrum activity (Rodríguez-Martínez et al., 2010). ADC-56 possesed an R148Q mutation also within the Ω loop, that enabled the enzyme to hydrolyze cefepime (Tian et al., 2011). Thus, the G246S mutation within the Ω loop of the AmpC from AC29 and AC30 could be responsible for the extended spectrum activity. This could be examined and verified by site-directed mutagenesis of the pET30a recombinant clones. Likewise, the contribution of the R80S mutation toward extended spectrum activity should be investigated even though it is located in a non-active site.

## Conclusions

In this study, we presented the comparative genome analyses of two Malaysian *A. baumannii* strains AC29 and AC30 that belonged to the ST195 lineage and had identical *Apa*I pulsotype but different susceptibilities to polymyxin. Their MLST profiles and phylogenetic clustering based on their concatenated MLST sequences clearly showed these strains belonging to the International Clone II (IC-II) group. Novel resistance island (RI) variants and plasmids were discovered from the genome sequence of these strains. Both strains shared a similar AbaR4-type RI of approximately 22 kb interrupting the *comM* gene insertional hotspot and which contains the carbapenem resistance gene, *bla*_OXA−23_ within a composite transposon, Tn*2006*. The island also encodes genes conferring resistance to tetracyclines, sulphonomides and streptomycin. Both *A. baumannii* strains harbored a small ~8 kb cryptic plasmid which encode putative virulence determinants (TonB-dependent receptor and septicolysin) as well as a XerC/XerD recombination site. Plasmid pAC30b is found only in AC30 but not AC29 and contained the Tn*1548::armA* island that confers resistance to aminoglycosides and macrolides. Interestingly, this island was found in the chromosome of AC29. Both AC30 and AC29 harbored a ~70 kb conjugative plasmid designated pAC30c and pAC29b with pAC29b containing a copy of *bla*_OXA−23_. Thus, genomic islands and, to a lesser extent, conjugative plasmids, appeared to play an important role in the dissemination and acquisition of antibiotic resistance determinants in the Terengganu *A. baumannii* strains. Experimental evidence also indicated that polymyxin resistance in AC30 may have developed through a combination of *pmrAB* upregulation and partial impairment of the lipopolysaccharide layer. The *bla*_AmpC_ variant encoded by both AC29 and AC30 was also shown to be likely an extended-spectrum *Acinetobacter*-derived AmpC (ESAC) conferring resistance to cefepime as well as imipenem. Whole genome sequencing of the two Terengganu *A. baumannii* clinical strains and subsequent experiments enabled a detailed characterization of their genetic repertoire of resistance, thereby giving us an insight into the genetic blueprint of Malaysian isolates of this increasingly important and deadly nosocomial pathogen.

### Conflict of interest statement

The authors declare that the research was conducted in the absence of any commercial or financial relationships that could be construed as a potential conflict of interest.
